# Discovering Structural Motifs in miRNA Precursors from the *Viridiplantae* Kingdom

**DOI:** 10.3390/molecules23061367

**Published:** 2018-06-06

**Authors:** Joanna Miskiewicz, Marta Szachniuk

**Affiliations:** 1 Institute of Computing Science, Poznan University of Technology, 60-965 Poznan, Poland; joanna.miskiewicz@cs.put.poznan.pl; 2 Institute of Bioorganic Chemistry, Polish Academy of Sciences, 61-704 Poznan, Poland

**Keywords:** miRNA biogenesis, structural patterns, DCL1

## Abstract

A small non-coding molecule of microRNA (19–24 nt) controls almost every biological process, including cellular and physiological, of various organisms’ lives. The amount of microRNA (miRNA) produced within an organism is highly correlated to the organism’s key processes, and determines whether the system works properly or not. A crucial factor in plant biogenesis of miRNA is the Dicer Like 1 (DCL1) enzyme. Its responsibility is to perform the cleavages in the miRNA maturation process. Despite everything we already know about the last phase of plant miRNA creation, recognition of miRNA by DCL1 in pre-miRNA structures of plants remains an enigma. Herein, we present a bioinformatic procedure we have followed to discover structure patterns that could guide DCL1 to perform a cleavage in front of or behind an miRNA:miRNA* duplex. The patterns in the closest vicinity of microRNA are searched, within pre-miRNA sequences, as well as secondary and tertiary structures. The dataset consists of structures of plant pre-miRNA from the *Viridiplantae* kingdom. The results confirm our previous observations based on *Arabidopsis thaliana* precursor analysis. Hereby, our hypothesis was tested on pre-miRNAs, collected from the miRBase database to show secondary structure patterns of small symmetric internal loops 1-1 and 2-2 at a 1–10 nt distance from the miRNA:miRNA* duplex.

## 1. Introduction

MicroRNAs (miRNAs) represent a group of small noncoding RNAs (sRNA) that consist of about 21–24 nucleotides [1,2,3,4,5,6,7,8]. They are present in animals, plants, and single-cell eukaryotes. The key role of miRNA is to regulate gene expression via degrading or blocking the targeted mRNA transcript [9,10]. With the ability to silence various genes, microRNA can modulate the homeostasis of the organism by interfering with specific mRNAs, as well as by preventing further expression of genes engaged in development, metabolism, or differentiation [3,11,12,13,14]. Mis-regulation of miRNAs, which are involved in different biological processes, is believed to be a major contributor to various diseases [15]. The recognition of targeted transcripts comes through nearly complete (in plants) or partially complete (in animals) base pair complementarity [6,16]. The multistep miRNA biogenesis differs between plants and animals, mainly in the cell location where each stage of the process is held and in the contributing proteins. The transcribed miRNA gene (pri-miRNA) in animals is cleaved into a precursor (pre-miRNA) structure by a microprocessor. The microprocessor primarily consists of two enzymes: RNAse III Drosha and DiGeorge Syndrome Critical Region 8 (DGCR8) (in several organisms DGCR8 is replaced by Pasha) [17,18,19]. At this phase, pre-microRNA is transported from the nucleus to the cytoplasm by Exportin 5 protein (XPO5). Next, Dicer (the other RNase III type enzyme), performs cleavages in pre-miRNA to release the duplex of microRNA (miRNA:miRNA*) [19]. In plants, all endonucleolytic cleavages of pri-miRNA and pre-miRNA are performed in the nucleus by Dicer-Like 1 (DCL1), being a homologue of Dicer. The process of plant miRNA maturation also requires engagement of HYPONASTIC LEAVES 1 (HYL1), a protein that contains a dsRNA-binding domain, and SERRATE (SE), a protein containing a zinc-finger domain. After creation, pre-miRNA is exported to the cytoplasm by the HASTY enzyme, a homologue of XPO5 [5,12,20,21]. In both, animal and plant cells, the miRNA:miRNA* duplex consists of a guide and a passenger strand. During incorporation of the duplex into the RNA-induced silencing complex (RISC), the passenger strand is discarded, while the guide strand leads the complex toward the target mRNA [22,23,24]. The passenger strand (miRNA*) is either degraded or used as a guide for other transcripts. Besides miRNA, which determines the targeted mRNA via base pair complementarity, RISC includes an ARGONAUTE (AGO) protein, the effector molecule with slicing activity [7,25]. The RISC enables degradation of the target mRNA or inhibition of the translation process by several mechanisms, including ARGONAUTE endonuclease activity, which enables slicing of targeted mRNA [3,5,10,25]. Biogenesis of animal miRNAs can be classified as a well-known process. The cleavages performed on animal pre-miRNA by the molecular ruler Dicer are measured from the pre-miRNA terminus, either the 3′ or the 5′ end, to the RNase III domain-dependent cleavage site [26,27]. In plants, it is still a mystery how the DCL1 enzyme recognizes miRNAs within pre-miRNA structures to perform cuts and release the miRNA:miRNA* duplex. Therefore, we have decided to analyze a set of available pre-miRNA structures and look for structural patterns occurring in miRNA vicinity. It is assumed that some motifs should exist and guide DCL1. Herein, we present a broad approach to pattern searching within pre-miRNAs. We have applied it to structures from four phyla of the *Viridiplantae* kingdom. We drew from our previous research concerning structural motifs in precursor microRNAs of *Arabidopsis thaliana*.

## 2. Results

### 2.1. A Scheme of Data Processing

**Figure 1:**
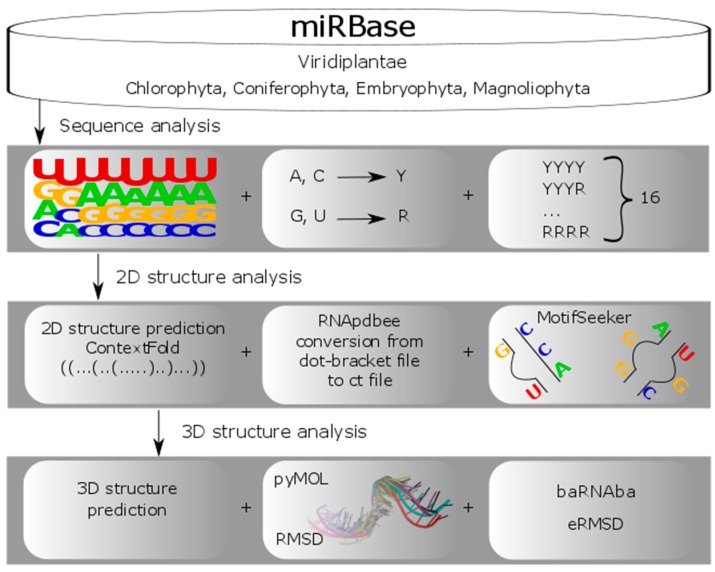
Precursor microRNA (pre-miRNA) analysis workflow.

Our research project has followed several steps (Figure 1). At first, the data for an analysis was collected and pre-processed. After dataset preparation, a semi-automated processing of pre-miRNAs followed. It was conducted at three structure levels. We started by investigating the sequences, and going through secondary structure studies, we ended up with a three-dimensional (3D) structure analysis. A detailed description of these steps is provided in the following paragraphs.

### 2.2. Dataset Preparation

In order to find structural motifs in plant pre-miRNA, which could help understand DCL1 performance, we prepared a dataset based on sequences stored in the miRBase database [4]. We considered records under the *Viridiplantae* kingdom assigned to the following phyla: *Magnoliophyta* (6547 sequences), *Coniferophyta* (108 sequences), *Chlorophyta* (50 sequences), and *Embryophyta* (287 sequences). Altogether, our initial collection contained 6992 sequences. The Table S1 from Supplementary Materials contains number of sequences extracted from miRBase website [4] distributed by phylum, clade, family and species. Next, we extracted the relevant information of collected *Viridiplantae* from the miRBase [4] website, and shaped it to adjust to further processing. This was done using self-prepared scripts written in Python language. The prepared data files contained an accession number for each pre-miRNA (in accordance with the miRBase nomenclature) assigned to the sequence, and an miRNA position within its appropriate precursor. From the miRBase [4] database, we also collected evidence about every miRNA found within the set of 6992 sequences, which could be experimental (by similarity) or not experimental. In our research, we planned to focus the analysis on the miRNA vicinity. Thus, we needed to have the sequences and structures of miRNA precursors containing miRNAs with sufficiently large neighbouring regions. It had been decided that eight nucleotides per strand constituted a sufficient size for the vicinity sequence to be analyzed. In the initial collection of 6992 sequences, we identified 5345 pre-miRNA sequences in which miRNAs were surrounded by at least 8 nt on their 5′ and 3′ ends: 4956 from *Magnoliophyta*, 80 from *Coniferophyta*, 38 from *Chlorophyta*, and 271 from *Embryophyta*. These sequences were selected to form the basic *S8* set used in the majority of forthcoming experiments. Within this set, at least one miRNA per each sequence was confirmed experimentally (in the subset of 4388 sequences) or by similarity (within the subset of 343 sequences). In the remaining 614 sequences of the *S8* set (<11.5%), miRNAs were confirmed non-experimentally (i.e., the miRNA sequence was revealed by sequencing, and not used in any experiment yet).

Further, we found it also necessary to limit the miRNA vicinity size to 4 nt. To meet this requirement, from the initial 6992 sequences, we picked 5975 pre-miRNAs with at least 4 nucleotides on both sides of miRNA: 5555 from *Magnoliophyta*, 99 from *Coniferophyta*, 41 from *Chlorophyta*, and 280 from *Embryophyta*. These were collected in the *S4* set, which included 5345 sequences from the *S8* set (vicinity size ≥8 nt) and 630 sequences with vicinity size between 4 and 7 nt. These sequence collections allowed us to properly define the search space for our computational experiments. Within the *S4* set, at least one miRNA per sequence was confirmed experimentally (in the subset of 4890 sequences) or by similarity (within the subset of 389 sequences). In the remaining 696 sequences of the *S4* set (<12%), miRNAs were not confirmed experimentally (i.e., miRNA sequence was revealed by sequencing, and not used in any experiment yet).

### 2.3. Primary Structure-Based Analysis

**Figure 2:**
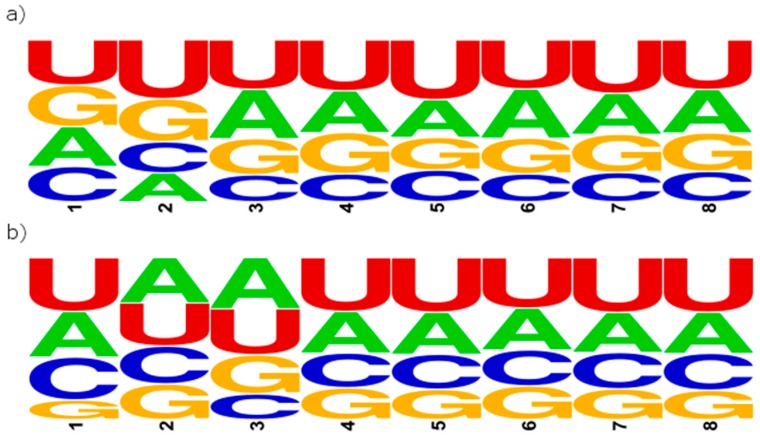
WebLogo 2.8.2 [28] diagram for sequences from the (**a**) *VS8-5′* and (**b**) *VS8-3′* subsets.

In the first computational experiment, we have used the *S8* set of the pre-miRNA sequences. In every sequence from *S8*, either one or two miRNAs were found. We identified an 8 nt-long vicinity sequence on the 5′ and 3′ end of each of these miRNAs. These sequence fragments were extracted to form *VS8-5′* and *VS8-3′* subsets of a large *VS8* collection, including 12802 vicinity sequences with the length equal to 8 nt exactly. Subset *VS8-5′* contains 6401 vicinity sequences occurring in the miRNA vicinity on the 5′ end, and subset *VS8-3′* has 6401 sequences from the 3′ end vicinity. Both subsets, *VS8-5′* and *VS8-3′*, were processed using WebLogo tool versions 2.8.2 (https://weblogo.berkeley.edu/logo.cgi) [28] and 3.0 (http://weblogo.threeplusone.com/create.cgi) [28]. WebLogo allowed us to obtain a diagram showing the most- and the least-frequent nucleotides occurring on each of the eight positions of miRNA vicinity sequence. The first position in each sequence is the first nucleotide behind the microRNA, counting towards the 3′ end (in the *VS8-5′* subset) or towards the 5′ end (in the *VS8-3′* subset). The most frequent nucleotides are shown at the top of the stack, while the least frequent ones are at the bottom (Figure 2). Detailed information about nucleotides occupying the following positions within vicinity sequences is provided in Table 1 (for the *VS8-5′* subset) and Table 2 (for the *VS8-3′* subset).

It can be observed that Uracil is the most frequent nucleotide on almost every position of each vicinity sequence. In sequences from the *VS8-5′* subset, the second position is heavily occupied by Uracil (36.38% of sequences in *VS8-5′* have Uracil on the second position), and rather poorly by Adenine (17.78%). This can indicate an unpairing in the structure, which occurs exactly on this position. In the *VS8-3′* subset, bigger differences are observed between Cytosine and Guanine occupation. The biggest difference reaches 14.22%, and concerns the first position of the vicinity sequence. In the *VS8-5′* subset, nucleotides on the first position are almost evenly distributed, while the second position seems to create an unpaired region. The *VS8-3′* subset seems to be contrary to this. It shows almost equally distributed values on the second position and highly varied distribution in the first position. Thus, it is possible that in the region of the first two positions beyond the miRNA sequence, one could find a small mismatch, revealed as a bulge or a loop in the structure. In the second experiment, aimed to search for sequential motifs in miRNA vicinity, we decided to represent each nucleotide in nucleotide ambiguity code (IUPAC) [29], based on the number of carbon-nitrogen rings, as a purine (R) or pyrimidine (Y). At first, this experiment was run on the previously created *VS8-3′* and *VS8-5′* subsets. In every vicinity sequence from these subsets, we changed the representation of adenines (A) and guanines (G) into purines (R) and uracils (U) and cytosines (C) into pyrimidines (Y). Next, we searched for exactly 8 nt-long patterns that were also encoded using the two-letter alphabet {R, Y}. All permutations for eight positions with two possible variants, purine or pyrimidine, gave us 256 possible patterns. We did not observe any significant results in this experiment. Therefore, we decided to restrict the search space and run the experiment for shorter vicinity sequences. We have taken the *S4* set of 5975 pre-miRNAs, containing miRNAs with neighbouring regions having at least 4 nucleotides on both the 5′ and 3′ end next to the miRNA region. From this collection, we extracted 14300 vicinity sequences 4 nt long, and divided them into two subsets, *VS4-5′* and *VS4-3′*, in the same manner as *VS8*. Each of these subsets contained 7150 short sequences. Every vicinity sequence from *VS4-5′* and *VS4-3′* was next represented with the two-letter alphabet {R, Y}, and the search for 4 nt-long patterns was performed, providing the results as presented in Table 3.

The first symbol of a pattern corresponds to the nucleotide on the first position beyond miRNA sequence. From these statistics, we can observe that five of the most frequent motifs start with pyrimidine: YYRY, YRRR, YRRY, YYYY, and YYRR. This suggests that many sequences which encounter miRNA involve uracil or cytosine right before the first nucleotide of miRNA sequence.

### 2.4. Secondary Structure-Based Analysis

**Figure 3:**
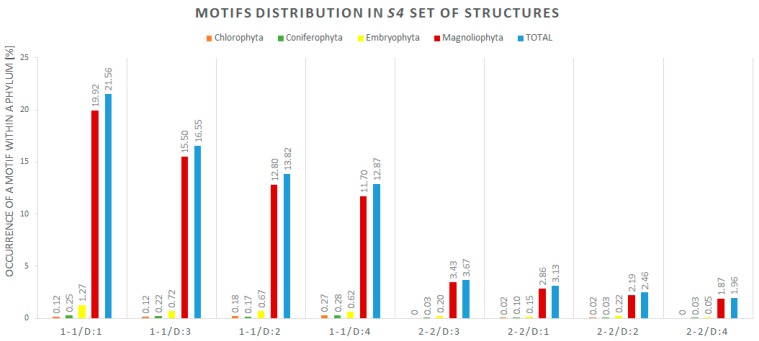
Distribution of eight most-occurring two-dimensional (2D) motifs in 5975 structures by phyla. The results are arranged from the least frequent motif to the most common one.

The second part of our analysis concerned the secondary structures. Since our input data collection contained sequences only, we decided to predict their secondary structures using ContextFold version 1.0 [30] installed on a local computer. The software was chosen based on the CompaRNA benchmark [31]. All 5975 sequences from the *S4* set were processed by ContextFold [30] to predict their secondary structures. Predicted structures were encoded in dot-bracket notation. For the facilitation of further analysis, we used RNApdbee program (http://rnapdbee.cs.put.poznan.pl/) [32,33,34] to transform two-dimensional (2D) structures from dot-bracket to CT (Connect) format. Next, we applied a script called MotifSeeker implemented in Python language. The MotifSeeker processes CT files, and searches for bulges and internal loops in the vicinity of the miRNA:miRNA* duplex (up to four nucleotides beyond the miRNA on both sides). The generated output file contains brief information about what motif has been found, on which strand, and how far it was from the microRNA. Guided by our previous study of the pre-miRNA sequences of *Arabidopsis thaliana* [5] and current WebLogo [28] results, we expected an accumulation of mismatches between the first and fourth position beyond miRNA. Although it is known that similar sequences do not always maintain the similarities at higher structural levels [35], we supposed that in our case, the analyzed structures would share some of their pattern in the short fragment beyond the miRNA:miRNA* duplex at the secondary or tertiary structural level. MotifSeeker allowed us to identify the most frequently occurring secondary structure pattern, along with its distance from the miRNA:miRNA* duplex, and a number of structures in which the motif was found. According to our assumptions, the first eight most frequent patterns had small mismatches: symmetric internal loops 1-1 (single unpaired nucleotide on every strand of the vicinity region) and 2-2 (two unpaired nucleotides on every strand of the vicinity region). We have found that in 21.56% of the 5975 secondary structures, the first nucleotides beyond the miRNA:miRNA* duplex were unpaired and formed symmetric 1-1 internal loops. The same 1-1 pattern was shared by 13.82% of the secondary structures, starting from the second position, and 16.55% of the structures starting from the third position beyond the miRNA:miRNA* duplex. This means that over 50% (exactly 51.93%) of the analyzed secondary structures contain the 1-1 motif at the maximum distance of three positions beyond miRNA. In Table 4, we present the exact number of motifs found within the structures in which we discovered the pattern. All motifs identified by MotifSeeker are represented in Figure 3, where each position is defined by the pattern type (1-1 or 2-2) and the distance between the motif and the miRNA, from 1 nt (D:1) up to 4 nt beyond miRNA (D:4). The MotifSeeker code and input files can be found here: http://bio.cs.put.poznan.pl/fileserver/.

### 2.5. Tertiary Structure-Based Analysis

**Figure 4:**
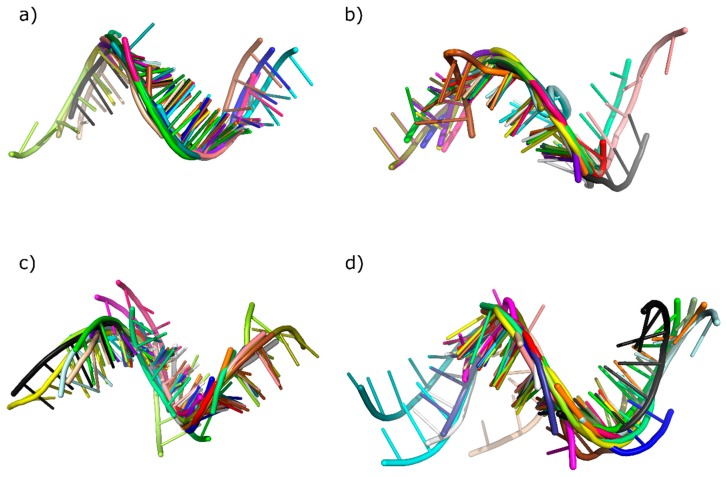
Aligned three-dimensional (3D) substructures within each phylum: (**a**) *Chlorophyta*, (**b**) *Coniferophyta*, (**c**) *Embryophyta*, and (**d**) *Magnoliophyta*.

In the third stage of analysis, the tertiary structures of miRNA vicinity were analyzed using bioinformatics tools. Over many years, lots of methods for RNA 3D structure analysis have been developed [36,37]. In our experiments, we decided to focus on three of them: RNAComposer [38,39], PyMOL [40], and baRNAba [41]. First, we predicted 40 tertiary structures by using RNAComposer [38,39]. The input set for the prediction process included 10 sequences for each phylum picked randomly from S4 dataset. The obtained models were next processed by using the PyMOL program [40]. From each predicted tertiary structure, the closest vicinity regions of miRNA were cut out for alignment. Due to the shift between the 5′ and 3′ miRNA, we decided to use regions that were overlapping the miRNA:miRNA* duplex for 4 nt beyond the duplex and 4 nt within the duplex. This resulted in obtaining 8 nt-long structures from both sides of the miRNA:miRNA*. For each phylum, we have generated 20 short 3D fragments. Among them, one random structure was chosen as a reference—the remaining ones were aligned to it. Thus, we created four different alignments (Figure 4), with root mean square deviation (RMSD) values measured by PyMOL [40] and eRMSD values computed by the baRNAba software [41]. RMSD allowed us to measure the similarity between the superimposed atomic coordinates [42] whereas eRMSD facilitated to measure the distance between structures based only on the relative positions and orientations of nucleobases [41].

The RMSD values presented in Table 5 do not exceed 2.5 Å, while the average values are not higher than 1.5 Å. Relatively low values are also found in Table 6, representing eRMSD. The highest value in Table 6 is 1.101 Å, and all four calculated averages are below 0.90 Å. In both situations, the results indicate high 3D structure similarity between the four phyla. Thus, the closest region to the miRNA:miRNA* duplex seems to be highly conserved between the phyla in *Viridiplantae* kingdom.

## 3. Discussion

MicroRNA research has become increasingly popular since these molecules were discovered [43,44]. Nowadays, it is not only in-vivo or in-vitro methods that are used to examine the nature of miRNAs. In-silico approaches allow us to predetermine the direction of experiments, and help to narrow the search space to answer the questions raised. Here, we focused on plant microRNAs and performed a series of computational experiments using bioinformatic methods and programs. At each level of the RNA structure, we searched for specific motifs that could guide the DCL1 enzyme to the cutting position of the miRNA:miRNA* duplex. Every analytical step we carried out led to us finding small mismatches placed in the closest vicinity of the 5′ and 3′ ends of the miRNA. Although the results of sequence analysis did not unequivocally indicate the specific unpairing in this area, the secondary structure study proved this hypothesis. In the phase of 2D structure analysis, we discovered a high number of symmetric 1-1 and 2-2 internal loops occurring no further than four nucleotides behind the miRNA:miRNA* duplex. This supports the results of our previous research on *Arabidopsis thaliana*, where we also found a significant number of such motifs in the direct vicinity of miRNA [10]. Additionally, we examined tertiary structures by aligning predicted 3D models of the miRNA neighbourhood and calculating two distance measures (RMSD and eRMSD) between them, divided by phyla. The results confirmed the appearance of a conserved region close to the duplex. In conclusion, the taken bioinformatic pathway helped us to discover potential motifs recognized by the DCL1 enzyme. By examining each structural level, we managed to extract the necessary information and draw proper conclusions. Obtained via in-silico methods, the results clearly point out the significance of closest vicinity of miRNA and mismatches occurring in this region.

## 4. Materials and Methods

The research focused on three structural levels of RNA architecture: sequence, secondary, and tertiary structure. Sequences were obtained from miRBase (http://www.mirbase.org/), a repository of pre-microRNAs of various organisms [4]. Based on experimental data, this database includes not only sequences, but also positions of miRNA on the 5′ and 3′ strand. Annotation and sequence data for each entry are displayed on the website, along with the proposed secondary structure model of the pre-miRNA.

### 4.1. WebLogo

Sequence analysis was performed using WebLogo [28], aimed to discover the most frequent nucleotide on each position of miRNA vicinity area. WebLogo version 2.8.2 [28] (https://weblogo.berkeley.edu/logo.cgi) produced diagrams showing the frequency of nucleotides at each analyzed position. The first position is marked as the closest one to miRNA. WebLogo version 3.0 [28] (http://weblogo.threeplusone.com/create.cgi) was used to generate numerical values of nucleotide frequencies. WebLogo 2.8.2 [28] was used with the following settings for image format and size: *Image format* as eps (vector), and Logo Size per line equals to 18 × 5 cm. For advanced logo options, the settings were as follows: *Sequence Type* was automatic detection; *First Position Number* was 1; *Small Sample Correction* was true; *Frequency Plot* was true; *Logo Range* was none; *Multiline Logo (Symbols per Line)* was false. The advanced image options were set as follows: *Bitmap Resolution* at 96 pixels/inch (dpi); *Antialias Bitmaps* was set to true; *Title* was none; *Y-Axis Height* was none; *Show Y-Axis* was true; *Show X-Axis* was true; *Y-Axis Label* was none; *X-Axis Label* was none; *Show Error Bars* was false; *Boxed/Boxed Shrink Factor* was false; *Show Fine Print* was true; *Label Sequence Ends* was false; *Outline Symbols* was false; and *Y-Axis Tic Spacing* was 1 bit. Colors settings were selected as default. In the WebLogo 3.0 tool [28], we used following parameters: *Title* was none; *Output Format* was data (plain text); *Sequence type* was auto; *Logo size* was medium; *Stacks per Line* was 40; *Ignore lower case* was false; *Units* were probability; *First position number* was 1; *Logo range* was none; *Figure label* was none; *Scale stack widths* was true; *Composition* was auto; *Error bars* were false; *Show Sequence Ends labels* was false; *Version Fine Print* was true; *X-axis* was true; *Y-axis* was true; *Y-axis scale* was auto; *Y-axis tic spacing* was 1.0; and *Color Scheme* was auto.

### 4.2. Purine–Pyrimidine Patterns

The next phase of the study required changes in miRNA vicinity sequences. Adenine and guanine were represented as R (which denotes purines), while cytosine and uracil were represented as Y (which denotes any pyrimidine). These substitutions were applied by self-created script in Python language. Again, sequence patterns were searched in the modified sequences with using self-developed Python script.

### 4.3. ContextFold

In the second analytical step, the secondary structures were predicted via the ContextFold program [30]. This program, installed on a local computer, produces files which contain 2D structures defined in dot-bracket notation. In this format, each unpaired nucleotide (mismatch or gap) is represented as a single dot, and a paired nucleotide as an opening or closing bracket. The command used, *java-cp bin contextFold.app.Predict in: input_file.txt out:output_file.txt*, enabled prediction of the secondary structures for all RNA sequences in the input file, using the (default) supplied StHighCoHigh trained model, and saving the result to the output file [45].

### 4.4. RNApdbee

To facilitate further research, we used the RNApdbee webserver [32,34] (http://rnapdbee.cs.put.poznan.pl/) to convert dot-bracket representation into CT format. The latter data format describes the position of nucleotide in the sequence, nucleobase encoding, the position of the previous and next nucleotides in the sequence, and the index of the paired nucleotide. If the nucleotide is unpaired, the index equals 0. On the RNApdbee website, we chose the third mode of analysis (i.e., third tab page, selecting “(….) → image”). After uploading the structures in dot-bracket notation, we selected the options to (1) identify the structural elements by treating pseudoknots as paired residues, and (2) visualize the secondary structure using the VARNA-based procedure. When the computation was finished, we downloaded the results in CT file format.

### 4.5. MotifSeeker

The secondary structures were examined by self-developed script named MotifSeeker. MotifSeeker reads CT files and additional information from the pre-miRNA id and its microRNA positions at the 5′ and 3′ ends. Next, the script searches for bulges and internal loops, providing information about the type of mismatch and its distance from miRNA.

### 4.6. RNAComposer

The last phase of our research involved the prediction of tertiary structures of RNA. We selected 10 secondary structures from each phylum, and used them to predict their 3D structures using RNAComposer (http://rnacomposer.cs.put.poznan.pl/), running it in batch mode [38,39]. RNAComposer allows us to automatically predict tertiary RNA structures, up to 500 nt per structure, based on their secondary structure in dot-bracket format. It is possible for the user to choose one of the six secondary structure prediction methods incorporated into the system. For our analysis, we set the *Select secondary structure prediction method* option to “true”, and from the drop-down list we chose the ContextFold method [30]. The same can be done in the interactive mode of RNAComposer, where the user can either select the secondary structure prediction method by selecting it from drop-down list or by typing the method name in the next line after the sequence (no dot-bracket notation is required in this case), e.g.,: 

#zma_MIR168a >example GAAGCCGCGCCGCCUCGGGCUCGCUUGGUGCAGAUCGGGACCCGCCGCCCGGCCGACGGGACGGAUCCCGCCUUGCACCAAGUGAAUCGGAGCCGGCGGAGCGA ContextFold

Since we have used the batch mode, we could generate more than one 3D structure per secondary structure input. However, we decided to generate a single 3D structure model, and the *Maximum number of generated 3D models* was set to 1.

### 4.7. PyMOL

The obtained 3D structures were processed in PyMOL [40]. PyMOL software enables molecular visualization, measurement, processing, and model comparison. We used it to align structures within each phylum, and to measure the RMSD values between them. RMSD (root mean square deviation) is one of the standard measures that calculates an average distance between the atoms. 

### 4.8. BaRNAba

Finally, the baRNAba tool was applied to calculate eRMSD values, which refer to the distance considering only the relative positions and orientations of nucleobases [46]. The command applied for baRNAba tool was *./baRNAba --name output_file.txt ERMSD --pdb reference.pdb -f 1_structure.pdb 2_structure.pdb ... 19_structure.pdb*.

## Figures and Tables

**Table 1:** WebLogo 3.0 [28] results for vicinity sequences in the *VS8-5′* subset.

Position	A [%]	C [%]	G [%]	U [%]	R [%]	Y [%]
1	24.48	21.87	25.62	28.03	50.10	49.90
2	17.78	19.28	26.56	36.38	44.34	55.66
3	30.32	16.67	22.37	30.64	52.69	47.31
4	25.68	17.31	25.46	31.54	51.15	48.85
5	23.18	19.56	20.26	36.99	43.45	56.55
6	30.21	17.23	22.11	30.45	52.32	47.68
7	25.17	18.12	23.54	33.17	48.71	51.29
8	26.71	18.75	23.76	30.78	50.48	49.52

**Table 2:** WebLogo 3.0 [28] results for vicinity sequences in the *VS8-3′* subset.

Position	A [%]	C [%]	G [%]	U [%]	R [%]	Y [%]
1	27.98	26.51	12.19	33.32	40.17	59.83
2	27.90	23.37	22.09	26.64	49.99	50.01
3	31.48	15.92	24.14	28.46	55.62	44.38
4	25.56	21.45	19.72	33.28	45.27	54.73
5	25.84	21.67	18.73	33.76	44.57	55.43
6	25.73	22.26	20.81	31.20	46.54	53.46
7	24.57	22.54	19.00	33.89	43.57	56.43
8	25.31	22.81	18.15	33.73	43.46	56.54

**Table 3:** Pattern occurrence in the *VS4-5′* and *VS4-3′* subset.

Pattern	*VS4-5′* [%]	*VS4-3′* [%]	Total [%]
RRYR	4.36	3.82	4.09
YRYR	4.41	4.57	4.49
RYYR	6.22	3.90	5.06
RRRY	5.43	5.92	5.67
RYRY	6.08	5.33	5.71
RRYY	6.13	5.30	5.71
YRYY	4.98	6.78	5.88
RYYY	6.90	4.98	5.94
YYYR	6.77	5.45	6.11
RYRR	7.50	5.29	6.39
RRRR	7.43	5.64	6.53
YYRY	6.77	6.67	6.72
YRRR	6.38	7.40	6.89
YRRY	4.83	10.10	7.46
YYYY	7.29	9.17	8.23
YYRR	8.55	9.68	9.11

**Table 4:** Motif occurrence in the *S4* set. The number of motifs was calculated based on the number of specific patterns in defined locations, referring to structures which contain at least one motif.

Motif/Distance	Number of Motifs	Number of Structures with at Least One Motif
1-1/D:1	1397	1288
1-1/D:3	1043	989
1-1/D:2	861	826
1-1/D:4	807	769
2-2/D:3	221	219
2-2/D:1	190	187
2-2/D:2	149	147
2-2/D:4	118	117

**Table 5:** RMSD values of 3D fragments from each phylum.

Fragment Id	RMSD [Å]			
	*Chlorophyta*	*Coniferophyta*	*Embryophyta*	*Magnoliophyta*
1	2.112	0.463	1.882	2.245
2	0.278	0.430	0.290	2.270
3	0.256	1.194	2.058	1.135
4	0.117	0.381	1.626	0.679
5	0.467	0.258	2.351	0.352
6	2.209	1.228	1.810	0.567
7	0.257	0.469	1.966	0.123
8	0.560	1.226	1.587	0.449
9	0.142	1.018	1.773	2.171
10	0.864	0.412	1.247	1.672
11	0.502	0.461	0.910	0.845
12	0.547	0.444	1.573	0.607
13	0.034	1.377	0.974	1.171
14	0.389	0.846	1.546	0.963
15	1.155	1.036	0.944	0.836
16	0.139	0.481	0.837	1.094
17	0.686	1.210	1.839	0.597
18	0.637	0.390	1.730	1.344
19	2.159	0.266	0.330	2.304
Average	0.711	0.715	1.435	1.128

**Table 6:** eRMSD values of 3D fragments from each phylum.

Fragment Id	eRMSD [Å]			
	*Chlorophyta*	*Coniferophyta*	*Embryophyta*	*Magnoliophyta*
1	0.459	0.765	0.802	0.554
2	0.788	0.771	0.434	0.503
3	0.587	0.436	0.725	0.730
4	0.291	1.047	0.776	1.101
5	0.477	1.047	0.868	0.325
6	0.432	0.746	0.858	0.444
7	0.561	1.025	0.868	0.832
8	0.442	0.799	0.817	0.365
9	0.459	0.675	0.767	0.455
10	0.438	0.800	0.842	0.643
11	0.386	0.749	1.080	0.390
12	0.251	0.753	0.841	0.398
13	0.605	0.745	0.906	0.457
14	0.410	0.680	0.791	0.447
15	0.410	0.891	0.883	0.394
16	0.463	0.729	0.901	0.467
17	0.564	1.023	0.788	0.331
18	0.528	1.058	0.764	0.604
19	0.453	0.712	0.810	0.604
Average	0.474	0.813	0.817	0.529

## Supplementary Materials

The following are available online. Table S1. Number of sequences extracted from miRBase website [4] distributed by phylum, clade, family and species.
