# Supplementary material for: Discovering Structural Motifs in miRNA Precursors from the Viridiplantae Kingdom
Source: Molecules. 2018 Jun 6;23(6):1367. doi: 10.3390/molecules23061367 (PMC6100135; doi:10.3390/molecules23061367)
Supplement: Supplementary file 1 [file molecules-23-01367-s001.pdf]

SUPPLEMENTARY MATERIAL

Phylum	Clade	Family	Species	Sequences
Chlorophyta			*Chlamydomonas reinhardtii (cre)*	50
Coniferophyta			*Cunninghamia lanceolata (cln)*	4
			Picea abies (pab)	40
			Pinus densata (pde)	29
			Pinus taeda (pta)	35
Embryophyta			*Physcomitrella patens (ppt)*	229
			Selaginella moellendorffii (smo)	58
Magnoliophyta			*Amborella trichopoda (atr)*	124
	eudicotyledons	*Araliaceae*	*Panax ginseng (pgi)*	29
		Asteraceae	*Cynara cardunculus (cca)*	48
			Helianthus annuus (han)	6
			Helianthus argophyllus (har)	3
			Helianthus ciliaris (hci)	3
			Helianthus exilis (hex)	2
			Helianthus paradoxus (hpa)	3
			Helianthus petiolaris (hpe)	3
			Helianthus tuberosus (htu)	16
		Brassicaceae	*Arabidopsis lyrata (aly)*	205
			Arabidopsis thaliana (ath)	325
			Brassica napus (bna)	90
			Brassica oleracea (bol)	10
			Brassica rapa (bra)	96
		Caricaceae	*Carica papaya (cpa)*	79
		Cucurbitaceae	*Cucumis melo (cme)*	120
		Euphorbiaceae	*Hevea brasiliensis (hbr)*	31
			Manihot esculenta (mes)	153
			Ricinus communis (rco)	63
		Fabaceae	*Acacia auriculiformis (aau)*	7
			Acacia mangium (amg)	3
			Arachis hypogaea (ahy)	23
			Glycine max (gma)	573
			Glycine soja (gso)	13
			Lotus japonicus (lja)	62
			Medicago truncatula (mtr)	672
			Phaseolus vulgaris (pvu)	8
			Vigna unguiculata (vn)	18
		Lamiales	*Avicennia marina (ama)*	2
			Digitalis purpurea (dpr)	13
			Rehmannia glutinosa (rgl)	32
			Salvia sclarea (ssl)	18
		Linaceae	*Linum usitatissimum (lus)*	124
		Malvaceae	*Gossypium arboreum (gar)*	1

			Gossypium herbaceum (ghb)	1
			Gossypium hirsutum (ghr)	78
			Gossypium raimondii (gra)	296
			Theobroma cacao (tcc)	82
		Ranunculaceae	*Aquilegia caerulea (agc)*	45
		Rhizophoraceae	*Bruguiera cylindrica (bcy)*	4
			Bruguiera gymnorhiza (bgy)	4
		Rosaceae	*Malus domestica (mdm)*	206
			Prunus persica (ppe)	180
		Rutaceae	*Citrus clementina (ccl)*	5
			Citrus reticulata (crt)	4
			Citrus sinensis (csi)	60
			Citrus trifoliata (ctr)	6
		Salicaceae	*Populus euphratica (peu)*	4
			Populus trichocarpa (ptc)	352
		Solanaceae	*Nicotiana tabacum (nta)*	162
			Solanum lycopersicum (sly)	77
			Solanum tuberosum (stu)	224
		Vitaceae	*Vitis vinifera (vvi)*	163
	monocotyledons		*Aegilops tauschii (ata)*	88
			Brachypodium distachyon (bdi)	317
			Elaeis guineensis (egu)	6
			Festuca arundinacea (far)	15
			Hordeum vulgare (hvu)	69
			Oryza sativa (osa)	592
			Saccharum officinarum (sof)	16
			Saccharum sp. (ssp)	19
			Sorghum bicolor (sbi)	205
			Triticum aestivum (tae)	116
			Triticum turgidum (ttu)	1
			Zea mays (zma)	172

Table 1S. Number of sequences extracted from miRBase website [3] distributed by phylum, clade, family and species.
